# The transformative impact of spirituality assessment on the quality of life for end of life cancer patients: a perspective paper

**DOI:** 10.3389/fpsyg.2026.1880381

**Published:** 2026-06-26

**Authors:** Federico Borgogni, Chiara Marzorati, Viktorya Voskanyan, Ricardo Pietrobon, Iris van der Heide, Augusto Caraceni, Norbert Couespel, Montse Ferrer, Mogens Groenvold, Stein Kaasa, Gennaro Ciliberto, Claudio Lombardo, Aude Sirven, Hugo Vachon, Galina Velikova, Alexandra Gilbert, Giovanni Apolone, Cinzia Brunelli, Gabriella Pravettoni

**Affiliations:** 1 Applied Research Division for Cognitive and Psychological Science, IEO, European Institute of Oncology IRCCS, Milan, Italy; 2 Department of Oncology and Hemato-Oncology, University of Milan, Milan, Italy; 3 SporeData OÜ, Tallinn, Estonia; 4 Netherlands Institute for Health Services Research (Nivel), Utrecht, Netherlands; 5 Dipartimento Di Scienze Cliniche e Di Comunità, Università Degli Studi Di Milano, Milan, Italy; 6 European Cancer Organisation (ECO), Brussels, Belgium; 7 Health Services Research Group, Hospital del Mar Research Institute, Barcelona, Spain; 8 CIBER en Epidemiología y Salud Pública, CIBERESP, Madrid, Spain; 9 Department of Medicine and Life Sciences, Universitat Pompeu Fabra, Barcelona, Spain; 10 Palliative Care Research Unit, Department of Geriatrics and Palliative Medicine GP, Copenhagen University Hospital—Bispebjerg and Frederiksberg, University of Copenhagen, Copenhagen, Denmark; 11 Section of Health Services Research, Department of Public Health, University of Copenhagen, Copenhagen, Denmark; 12 Department of Oncology, European Palliative Care Research Centre (PRC), Institute of Clinical Medicine, Oslo University Hospital, University of Oslo, Oslo, Norway; 13 IRCCS National Cancer Center “Regina Elena, ” on Behalf of DIGICOR, Rome, Italy; 14 Organisation of European Cancer Institutes (OECI), Brussels, Belgium; 15 Unicancer, Paris, France; 16 European Organisation for Research and Treatment of Cancer (EORTC), Quality of Life Department, Brussels, Belgium; 17 Leeds Institute of Medical Research at St James's, University of Leeds, Leeds, United Kingdom; 18 Fondazione IRCCS Istituto Nazionale Dei Tumori (INT), Milan, Italy; 19 Palliative Care, Pain Therapy and Rehabilitation Unit, Fondazione IRCCS Istituto Nazionale Tumori—Milano, Milan, Italy

**Keywords:** assessment, end of life, oncology, perspective, quality of life, spirituality

## Abstract

The global cancer burden is expected to increase dramatically in the coming years, providing considerable difficulties to healthcare systems around the world. While clinical practice frequently focuses on physical symptoms, there is a growing awareness that integrated, patient-centered care, particularly for patients at the end of life (EoL), can be critical for their wellbeing by addressing all aspects of their individual needs. This paper focuses on the essential role of spirituality as a component of quality of life across the EoL trajectory. Assessment of spiritual needs may have clinical value by providing patients with greater self-understanding and autonomy, allowing clinicians to propose humanized and targeted interventions, and guiding healthcare systems in optimizing resource allocation and economic sustainability. Despite its relevance, spiritual care is under-integrated into standard practices due to institutional barriers such as workload, insufficient staff training, and cultural values. To address these gaps, this paper presents the EUonQoL project as a model for developing culturally adapted, patient-centered assessment toolkits. This perspective argues that a comprehensive evaluation of spiritual wellbeing might be regarded as a therapeutic goal to ensure that end-of-life treatment matches with the individual's real priorities and needs.

## Introduction

The global impact of cancer is substantial, representing a major public health challenge that is expected to worsen, with an estimation of 35 million new cases projected by 2050 (Bray et al., 2024). Beyond epidemiological data, cancer also presents pervasive systemic challenges for healthcare systems in terms of economic management and clinical decision-making. These multifaceted challenges demand an integrated management strategy that extends beyond physical care and includes patient's psychosocial and overall wellbeing (Iliescu et al., 2024). Indeed, in response to the increasing global incidence of cancer, and in recognition of its consequences, significant research attention has been focused on improving standard interventions to equally address the clinical, physical, and psychosocial challenges associated with the disease. Nevertheless, clinical practice shows that integrated patient-centered care models have not yet been fully implemented due to persistent administrative and clinical barriers (Collaço et al., 2024). Such obstacles, such as limited communication between healthcare professionals, frequently result in the adoption of traditional symptom-oriented management as the default practice in many settings. Central to overcoming these implementation gaps is the need for a robust framework that captures the patient's subjective experience, most notably through the assessment of quality of life (QoL). The World Health Organization defines QoL as “an individual's perception of their position in life in the context of the culture and value systems in which they live and in relation to their goals, expectations, standards and concerns”, (The WHOQOL Group, 1995) highlighting individuals' own priorities, which vary depending on their personal history and surroundings. In this vein, social and cultural norms play a crucial role in shaping QoL, as they influence religious and spiritual beliefs, and personal relationship (Cucchi and Qoronfleh, 2025) by aligning individual needs with socially-structured values and meanings of life. While these overarching norms provide a consistent framework for meaning, the prioritization of specific needs often shifts as patients traverse through different stages of the oncological journey. Indeed, numerous studies point out how needs and values change between cancer phases (e.g., active treatment phase or palliative state) (Marzorati et al., 2025; Sala et al., 2025; Voskanyan et al., 2024). For instance, while during the active treatment patients may prioritize the therapies results or adherence, end of life (EoL) population may be more focused on spiritual peace, meaning, or existential concerns. Conversely, other factors, such as social support, remain relevant across all cancer phases, yet their function and significance dynamically change (Marzorati et al., 2025; Sala et al., 2025; Voskanyan et al., 2024). Specifically, early in the disease trajectory, social support is critical for overcoming immediate challenges and facilitating adaptation to the new condition (Zhu et al., 2024), whereas in the survivorship phase, its role may shift toward promoting autonomy and the development of new independence (Brinkman et al., 2018). Conversely, in EoL patients, social support becomes crucial for encouraging emotion expression, facilitating the addressing of existential concerns, and the achievement of mortality acceptance (Dobríková et al., 2016; Pun et al., 2023). Indeed, the sensitive nature of the EoL trajectory, especially after distressing cancer treatments, may shape the needs and perspectives of such patients, greatly affecting this delicate period of life.Besides demanding physical outcomes, this cancer phase challenges patients with the unpredictability of the prognosis, entailing a sense of hopelessness with related existential concerns (Tarbi and Meghani, 2019). EoL can lead to a shift in patients' perspectives and needs, reshaping their meaning of life as well as their social and individual wellbeing (Wajid et al., 2022). Even personal time perception changes, leading to a focus on day-to-day living (Rovers et al., 2019). In this context, when curative treatments prove no longer effective, oncological care should pivot to prioritize the patients' individual needs, transitioning the primary clinical goal toward the optimization of wellbeing and QoL. Sala et al. (2025) highlight that among the main factors affecting QoL in EoL patients, spirituality emerges as a critical component for individual needs. According to Mytko and Knight (1999), spirituality encompasses a feeling of connectedness with the self, community, and environment, striving for personal peace of mind and meaning of life. Scientific research shows that spiritual wellbeing is positively associated with QoL, as it helps mitigate anxiety and depressive symptoms, which are widely recognized as detrimental to individual wellbeing (Bai and Lazenby, 2015; Jetan et al., 2023). Coherently, spiritual wellbeing can offer significant protection against EoL despair in individuals facing imminent death, fostering greater acceptance of their condition (Pérez-Jiménez et al., 2026). This protective mechanism may be explained by spirituality's capacity in providing a sense of inner peace and harmony, and in helping individuals find meaning of life (Tirgari et al., 2022). If spirituality acts as a vital buffer against EoL despair, then its systematic evaluation could be considered as essential as the monitoring of physical symptoms. Coherently, the assessment of spiritual beliefs and values should be considered the very first step for paving the way for discussions and the eventual integration of this factor into clinical practice. Accordingly, this paper seeks to emphasize the essential role of understanding EoL patients' spiritual and cultural backgrounds, needs, and values in grasping their perspectives and designing effective interventions to enhance their QoL.

## How the assessment of spiritual outcomes could be clinically valuable?

In line with the outlined background, implementing assessment instruments to evaluate spirituality in EoL patients is crucial for a comprehensive understanding of their unique perspectives and wellbeing (Oliver et al., 2015). This nuanced understanding should allow for the design and application of patient-centered interventions across three distinct levels.

At a patient level, results of the assessment could serve as a quantifiable mirror, helping individuals objectify their status and gain insight into personal factors. Accordingly, a study on the emerging critical role of spirituality in EoL patients revealed that despite the majority of cancer patients and healthcare providers believe that routine spiritual care would positively impact patients, only 25% of patients had previously received such care, which indicates significant gaps in practice (Phelps et al., 2012). Integrating spirituality assessment into clinical settings may further support EoL patients in maintaining meaning, enhancing deeper and more tangible self-understanding. Indeed, enhanced self-comprehension and spiritual practice can motivate patients to foster better self-care (Mills et al., 2018). Coherently, scientific literature highlights that in the EoL stage this reflection enables patients to take greater control over their treatment and care plans, while supporting a proactive approach to change or manage their lives (Volker and Wu, 2011). A clearer understanding of their personal wellbeing, addressing individual needs, could guide EoL patients through this critical period of life and could improve their overall QoL (López-Salas et al., 2024).

Concurrently, at a clinician level, addressing this disparity should involve a deeper understanding of spiritual values, helping healthcare professionals build targeted and humanized interventions to improve patients' QoL while accounting for personal needs and desires. Scientific literature widely highlights the effectiveness of spiritual interventions on EoL cancer patients (Beglar et al., 2024). Indeed, spiritual care plays a crucial role in helping patients increase the motivation to manage life constraints and come to terms with their diagnosis, as spiritual support helps patients manage pain and attain spiritual peace (Beglar et al., 2024; Bacoanu et al., 2024). Hence, considering individual values as key care-drivers could enable the development of patient-centered interventions that align with patients' needs, thereby improving treatment outcomes (Oliver et al., 2015).

Finally, at a system level, the adoption of patient-centered questionnaires could drive structured, tailored interventions and their adoption around hospital facilities. The embracement of patient-centered care could enhance individual health outcomes while simultaneously lowering the cost of treatment for healthcare facilities (Pirhonen et al., 2020; Yu et al., 2023). This sustainable strategy could optimize resource allocation by preventing waste and ensuring spending is maximized where patient benefit is highest, directly supporting the economic sustainability of healthcare facilities. Indeed, scientific research demonstrate the efficacy of timely and integrated care in mitigating unnecessary expenditures within healthcare facilities while simultaneously enhancing patient outcomes (Rahman et al., 2018; Rocks et al., 2020). However, the implementation of such strategies and system changes may require time to be fully integrated, requiring a long-term vision to completely restructure the hospital departments responsible for palliative care management. The implementation of such well-defined strategies could further catalyze national healthcare reforms, bridging the gap for patients whose spiritual and personal needs are not yet integrated into standard EoL protocols. Hence, this approach could enable a more strategic macro-level management of healthcare resources, thereby fostering improved long-term health management across systems.

## Implementation barriers

Translating the above-mentioned systemic advantages into practice could require addressing significant institutional hurdles. Indeed, Laranjeira et al. (2023) identified several perceived barriers to providing spiritual care, with lack of time and work overload being particularly prominent. These findings suggest that the barriers could be related to understaffing and organizational and economic constraints. Indeed, the shortage of time, often perceived as a disregard of spiritual care delivery (Laranjeira et al., 2023), may indicate the level of under-consideration of the spiritual care in standard healthcare practices. Furthermore, the difficulty in training healthcare professionals to skillfully address spiritual needs contributes to the overlook of spiritual care, thus widening the existing gap (Batstone et al., 2020). Indeed, healthcare training remains a major obstacle to effective spiritual care, as provision can vary significantly based on the healthcare context and individual beliefs (De Luca et al., 2025).

**Figure 1:**
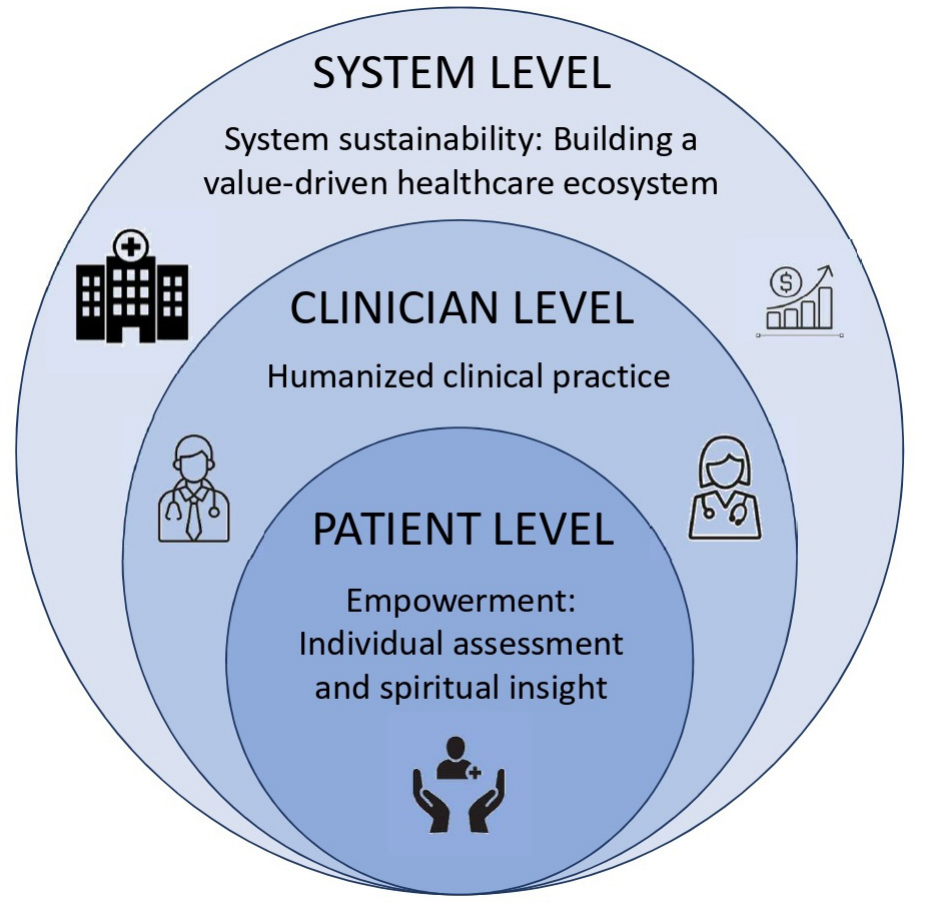
Impact of spiritual assessment on patient well-being and healthcare sustainability.

Integrating spiritual assessment into EoL care should be viewed as a multi-dimensional priority that influences personal experience, clinician's approach, and system's sustainability. These interconnected considerations are important at multiple levels, moving from individual self-understanding to broad healthcare reforms, as illustrated in Figure 1. Several implementation barriers have been highlighted above, underlining the actual difficulties in implementing changes in the EoL standard care. The risk of considering the implementation of these interventions without truly recognizing them as valuable is concrete. Therefore, the successful integration of spiritual assessment in EoL care requires a cultural shift within the health-care systems, to ensure that spiritual care could be genuinely considered meaningful and clinically relevant at multiple levels.

## How to improve the assessment of spiritual needs in EoL cancer patients? The EUonQoL project's proposal

Spirituality assessment tools are crucial in identifying EoL patients' spiritual needs, which can in turn significantly impact their QoL (Beglar et al., 2024; Sun et al., 2021). Ensuring the efficacy of such assessments could require the design of instruments that are not only patient-centered, but also deeply attuned to the cultural nuances that shape an individual's spiritual outlook. Indeed, spirituality is often part of an individual's culture, deeply shaping existential meaning and mental health features (Cucchi and Qoronfleh, 2025). A recent paper reports how cultural variable affect spiritual content in non-Westerns citizens by favoring spiritual explanations over mental health (Cucchi and Qoronfleh, 2025). Therefore, cultural differences may influence how spirituality is expressed, underlining the importance of culturally-sensitive assessment tools and approaches adoption. Coherently, several assessment instruments have been validated in the past years for the evaluation of spirituality in EoL patients, some of which considering cultural background (Monod et al., 2010; Munoz et al., 2015; Vivat et al., 2017). However, they still may need a progressive update over time in order to better account for evolving cultural values and ensure they accurately reflect the actual perspective of patients. Maintaining this alignment could ensure that the data collected remains clinically significant as societal and personal contexts shift. Hence, new assessment instruments should help patients and health-care professionals to gain a comprehensive understanding of all the relevant factors that shape personal values and QoL, thereby guiding practitioners in the development and adoption of tailored and personalized interventions.

The EUonQoL European project can represents a model of a newly designed, culturally-adapted, and patient-centered toolkit, capable of capturing patients' QoL while respecting individual beliefs and needs in light of their cultural identity (Apolone et al., 2024; Apolone and Brunelli, 2023). This initiative aims to develop, test, and validate the European Oncology Quality of Life Toolkit (EUonQoL-Kit), a unified, patient-centered instrument for assessing QoL along the cancer pathway (i.e., in cancer survivors, patients undergoing treatment, and EoL patients) (Apolone et al., 2024; Apolone and Brunelli, 2023). Following a comprehensive literature review and a multi-stage co-designed development process involving a broad panel of experts and co-researchers, the EUonQoL-kit has been created and is currently undergoing final refinement (Apolone et al., 2024; Apolone and Brunelli, 2023; Engelaar et al., 2024, 2025). A consistent and structured collaboration with co-researchers (people who have experienced cancer, either as a patient or an informal caregiver) was implemented to ground the project in the patient's reality (Engelaar et al., 2024, 2025). This ensured that the final outcomes could effectively reflect the true needs and values of the target population. Through an extensive and systematic literature review, the project's working group identified the core domains essential to cancer patients' wellbeing; within this framework, spirituality was confirmed as a critical factor significantly impacting individual QoL (Marzorati et al., 2025; Sala et al., 2025; Voskanyan et al., 2024; Lizano-Barrantes et al., 2026). The EUonQoL-Kit has been developed by merging existing items coming from libraries (such as EORTC Item Library and EORTC CAT item bank) and newly designed items, including spirituality as one of the considered domain. This project would respond to the urgent need for patient-centered assessment tools that can effectively capture individual perspectives in all cancer phases, including EoL. Ultimately, the EUonQoL project incorporates strategic implementation and dissemination activities designed to catalyze collaborative research and ensure the widespread adoption of these patient-centered innovations across the global oncological community.

## Conclusion

In our view, the spiritual dimension of EoL patients should be reconsidered as a central component of EoL care, given its key role in shaping patients' QoL. Standard interventions could risk overlooking some individual perspectives; hence, patient-centered spiritual assessment approaches should be considered to properly capture and address patient individuality. Given all of the scientific literature highlighting the positive impact of spiritual wellbeing on EoL patients' QoL, more specific spiritual assessment and tailored interventions would be a game-changer in the patient's wellbeing. The EUonQoL project exemplifies how a co-designed, patient-centered approach could successfully inform the development of robust assessment protocols for both research and clinical practice. Finally, this article serves as a call to action for patients, healthcare providers, and policymakers to recognize and address spirituality as well as other individual factors that profoundly shape patients' wellbeing.

## Author's note

The present paper was written on behalf of the EUonQoL consortium.

## Data Availability

The original contributions presented in the study are included in the article/supplementary material, further inquiries can be directed to the corresponding author.
